# Improving the Recovery of Patients with Subacromial Pain Syndrome with the DAid Smart Textile Shirt

**DOI:** 10.3390/s20185277

**Published:** 2020-09-15

**Authors:** Guna Semjonova, Janis Vetra, Vinita Cauce, Alexander Oks, Alexei Katashev, Peteris Eizentals

**Affiliations:** 1 Department of Morphology, Faculty of Medicine, Riga Stradins University, LV-1010 Riga, Latvia; Guna.Semjonova@orto.lv (G.S.); janis.vetra@rsu.lv (J.V.); 2 Statistics Unit, Faculty of Medicine, Riga Stradins University, LV-1046 Riga, Latvia; vinita.cauce@rsu.lv; 3 Institute of Design Technologies, Riga Technical University, LV-1048 Riga, Latvia; aleksandrs.okss@rtu.lv; 4 Institute of Biomedical Engineering and Nanotechnology, Riga Technical University, LV-1048 Riga, Latvia; aleksejs.katasevs@rtu.lv

**Keywords:** subacromial pain syndrome, physiotherapy, rehabilitation, smart textile, textile sensors

## Abstract

Wearable technologies provide many possibilities for applications in medicine, and especially in physiotherapy, where tracking and evaluation of body motion are of utmost importance. Despite the existence of multiple smart garments produced for applications in physiotherapy, there is limited information available on the actual impact of these technologies on the clinical outcomes. The objective of this paper is to evaluate the impact of the Double Aid (DAid) smart shirt, a purely textile-based system, on the training process of patients with subacromial pain syndrome. A randomized controlled trial was performed where patients with subacromial pain syndrome had to perform the assigned training exercises while employing the DAid smart shirt system. The core point of each exercise was to perform a movement while holding the shoulders stationary. The smart shirt was designed to sense even slight shoulder motion thus providing the patient with feedback on the accuracy of the motion, and allowing the patient to adjust the movement. The appropriate muscles should be strengthened through an increased effort to control the shoulder motion. The recovery of patients using the feedback system at the end of the treatment was compared to that of a reference group through standardized tests—the Disabilities of the Arm, Shoulder, and Hand score (DASH score), Closed Kinetic Chain Upper Extremity Stability test (CKCUES test), and internal/external rotation ratio. The test group that used the DAid system demonstrated significantly better results of the performed tests for all applied outcome measures compared to the reference group (*p* < 0.001). An overall positive impact on the patient recovery was observed from the DAid smart shirt system when applied for rehabilitation training of patients with subacromial pain syndrome.

## 1. Introduction

Shoulder pain has large health care costs and a major impact on the health of affected individuals, including absence from work and disability [1,2,3,4]. Between 7% and 34% of adults suffer from shoulder pain at any given point of time [5], whereas the lifetime prevalence is up to 67% [6]. Subacromial pain syndrome (SAPS) is one of the most frequent diagnoses contributing to up to 40% of cases of shoulder pain in general practice [3,6,7].

The ability to control the orientation and movement of the scapula, the bone connecting the humerus with the clavicle, also known as the shoulder blade, is essential for patients with SAPS [8]; consequently, physiotherapy is generally the first line of management for SAPS [9]. Conventional physiotherapy methods, such as movement control exercises, and scapular stabilization, have been proven effective in reducing pain and improving the function of the shoulder [8,10,11]; however, a face-to-face appointment with a physiotherapist is required for feedback and monitoring of correct movement pattern [11]. The average number of required physiotherapist visits for SAPS patients is above 7, which accounts for roughly 60% of the mean healthcare cost of SAPS treatment [4].

In recent years, there has been a growing trend of wearable technologies for applications in personalized and clinical healthcare [12], which in combination with telemedicine can be used for distanced physiotherapy [13]. The information on motor functions collected from body movement tracking systems not only is crucial as feedback for movement control, but can also boost the motivation, potentially resulting in increased participation [14]. Additionally, this information can be used by doctors for recovery monitoring and exercise program adjustment. However, most of the typical movement tracking systems are complicated and ill-suited for adoption under home conditions by individuals with a wide range of technical knowledge and physical abilities. Consequently, smart garment systems have been considered a potential substitute for upper body movement tracking in rehabilitation [15,16]. Such systems do not require extensive training of the user, and they provide an unobtrusive movement monitoring as the sensors are part of the clothing [17]. Although numerous wearable systems have been developed for different therapeutic tasks, most are tested only under laboratory conditions and using healthy test subjects, and no systems are currently actively used in practice [16,18]. A comprehensive review of the development of wearables for physiotherapy in the last decade was done by Wang et al. [16], concluding that the vast majority of the existing wearable systems for physiotherapy are inertial measurement unit (IMU) and accelerometer-based, with only a few exceptions being purely textile systems, none of which were clinically tested [16]. Purely textile systems or smart garments have several advantages over their IMU/accelerometer based counterparts, including ease of use and low manufacturing costs. The challenge of creating a sensing system for physiotherapy is producing a system with potential benefits surpassing the disadvantage of production costs and inconvenience of the application when compared to the conventional training method.

The Double Aid (DAid) smart shirt system for upper body movement monitoring is part of the DAid smart garment collection, which includes such smart garment systems as smart socks for gait analysis and smart shirts for physiotherapy assistance, developed by the research team at Riga Technical University. The smart shirt system for movement monitoring has been previously demonstrated to be an objective and convenient device for shoulder girdle motion monitoring during advanced motor tasks [19]. However, there is a lack of evidence that the application of the smart textile shirt technology has an impact on objective testing results for patients with SAPS. The present research aimed to evaluate the impact of the DAid smart shirt system on the training process of patients with SAPS. A randomized controlled trial was performed where a test group was selected to perform assigned training exercises employing the DAid feedback system, while a control group went through a conventional SAPS rehabilitation process. At the end of the training program, patient recovery was evaluated through standardized tests—the Disabilities of the Arm, Shoulder, and Hand score (DASH score) [20], Closed Kinetic Chain Upper Extremity Stability test (CKCUES test) [21], and internal/external rotation ratio [22]. The test results demonstrated a statistically significant difference between both groups, with the DAid group performing better in all applied tests. To the knowledge of the authors, this is the first study to demonstrate a clear benefit from the application of a smart garment system in clinical practice. The results of this study suggest that the application of smart garments for physiotherapy training assistance for patients with SAPS has an overall positive impact and can greatly enhance the efficiency of the training, thus improving the recovery.

## 2. Materials and Methods

### 2.1. Smart Shirt System

**Figure 1:**
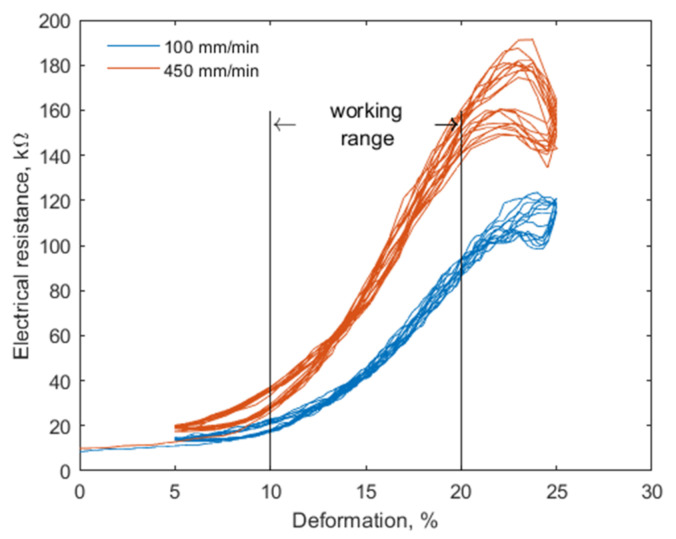
Textile sensor response for dynamic load at 100 and 450 mm/min deformation speed.

The DAid smart shirt system consists of two main parts, a tight fitness shirt with attached textile strain sensors and the data acquisition unit. The sensors were produced by incorporating a silver-coated conductive yarn into a cotton/elastomer base during the manufacturing process, resulting in a piezo-resistive material [23]. The electrical resistance is increased directly proportionally to the elongation of the sensor, which can be applied for the movement monitoring of the wearer through the deformation of the garment. Figure 1 demonstrates sensor response to stretching from 5% to 25% of the length, obtained by ElectroPuls™ E1000 (Instron Industrial Products, Norwood, MA, USA) dynamic test instrument at 100 and 450 mm/min deformation speed. The relatively low deformation speeds were chosen to represent slow, controlled movements during a physiotherapy training. It can be noted that the sensitivity increases with the deformation speed. The increased hysteresis at the end of the cycle is due to the relaxation of the sensor when the stretching is stopped. This relaxation depends on the deformation level and the speed of the movement before the steady state is acquired. It should be noted, however, that no significant deformations of the sensor should occur when an exercise is performed correctly, consequently, the sensors during the exercise are in the steady state; thus, the baseline, drift, and nonlinearity of the response plays no role in the performance of the system in general for this particular task.

**Figure 2:**
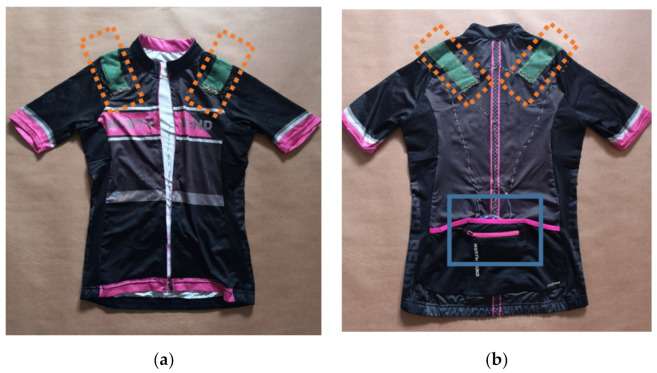
The Double Aid (DAid) smart shirt system ((**a**) front and (**b**) back) with a textile stretch sensor on each shoulder (marked by the dashed lines); the pocket on the back (marked by the blue line) is used for the data acquisition module.

The smart shirt prototype used in this study contained two textile sensors for movement monitoring (see Figure 2). The sensors were attached to a commercial elastane based fitness shirt with the elasticity comparable to that of the sensors by polychloroprene-based adhesive. The main requirements for the base shirt were comfort and elasticity for conforming to the shape of the body of the user. The sensors were attached to the shirt without pre-strain, but after putting on the shirt, the necessary strain (7–10%) to reach the linear working region is provided by the stretching of the shirt. The average calculated gauge factor in the working region of the sensor was 5–6.

The sensors were placed on the shoulder girdles on both sides, in the midpoint of *spina scapulae*, to the *angulus inferior scapulae*. The proper length, position, and orientation of the sensors were selected through trial and error. In the selected length and position, the sensors are the most sensitive to the shoulder elevation while being less sensitive to arm movements. The sensor configuration presented in this study, however, is by no means final and could be improved. In the general context of the rehabilitation, the sensor positioning depends heavily on the particular application and training plan. The placement of sensors is a crucial part of proper movement monitoring as incorrect placement can lead to insensitivity to the movement of interest or increased noise from the movement of other joints. The sensors in this configuration aim to inform the user about the movement of shoulders, which are supposed to be kept stationary during the training. When shoulders are moved, they either stretch or relax the fabric of the shirt in the direction of the movement, which in turn also deforms the stretch sensors. The sensors are oriented to be sensitive to shoulder protraction, retraction, and elevation. No calibration of the sensors is necessary as the user before each exercise has to stand in the initial position, which provides the user with a base-line measurement for each sensor. Previous research demonstrated that two sensors are sufficient for shoulder movement detection [17], and additional sensor benefit is negligible.

**Figure 3:**
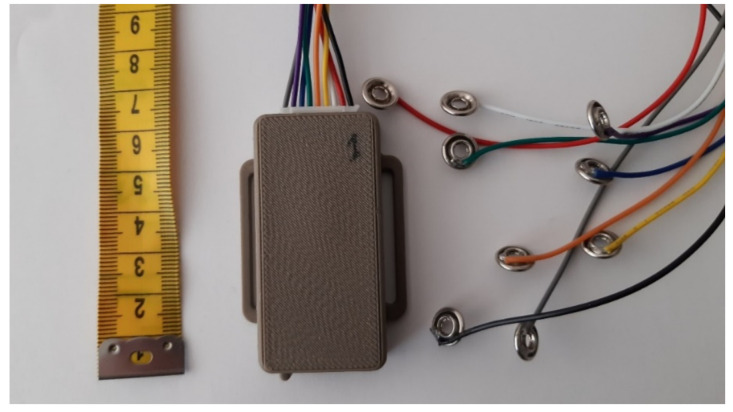
The data acquisition unit can measure up to eight channels and is connected to the system through textile snap fasteners. The unit measures only 6.6 cm × 4 cm × 1.3 cm and is positioned in a pocket on the back, and, thus, does not bother the user.

The sensors were connected to a single data acquisition unit through electroconductive yarns (Shieldex 117/17, Statex Produktions- und Vertriebs GmbH, Bremen, Germany) with low electrical resistivity (~1 kΩ/m) that were sewn manually [17]. The developed data acquisition unit (Figure 3) measured sensor electrical resistance at 175 Hz (maximal available for the used hardware) sampling rate was transferred via a Bluetooth connection to a computer and displayed to the user in real-time as a feedback of the movement. Considering that the physiotherapy exercises are relatively slow movements (<1 Hz), the applied sampling rate was sufficient for measuring the movements without loss of information.

### 2.2. Trial Design

A randomized controlled trial with parallel allocation using a 1:1 ratio (test group to control group size) was carried out in the “Orto Clinic” (Riga, Latvia), which specializes in traumatology, orthopedics surgery, and athlete rehabilitation. The study was performed between January 2019 and January 2020. Each participant was informed of the nature of the study and signed written informed consent for inclusion before participating in the study. The study was conducted following the Declaration of Helsinki and its later amendments or comparable ethical standards, and the study protocol was approved by the Ethics Committee of Riga Stradins University (6-3/39, 27.12.2018).

To provide randomization, SAPS patients to the physiotherapist involved in this study were appointed by an independent blinded administrator. Every even number patient, who matched the inclusion criteria, was included in the study group, while every odd number patient was included in the control group.

### 2.3. Participant Description

Patients aged 18–65 with a diagnosis of primary subacromial pain syndrome from an orthopedic specialist or rehabilitation doctor were included in the study. The inclusion criteria were:

- Non-traumatic shoulder problems that cause pain, localized around the acromion;
- Clinically set SAPS diagnosis by an experienced orthopedic specialist;
- Ultrasound or MRI imaging tests with a conclusion by an expert radiologist in shoulder pathology: subacromial impingement sign, subacromial pain syndrome;
- “Orto Clinic” patients that agreed to participate in study free of charge;
- At least 3 out of 5 SAPS tests should be positive: Painful arc, Empty can (Jobe test), External rotation resistance test, Hawkins–Kennedy test, Neer test [9,24,25].

The exclusion criteria were:

- Any radiologically verified malignancy;
- Previous fractures in the shoulder complex and/or shoulder surgery on the affected side;
- Osteoarthritis of the glenohumeral joint;
- Os acromiale or hooked III type acromion;
- Instability in any joint of the shoulder complex;
- Complete or partial rotator cuff or long head of biceps tendon tears;
- Clinically verified polyarthritis, rheumatoid arthritis, fibromyalgia, frozen shoulder;
- Symptoms from the cervical spine and pseudo paralysis;
- Any systemic or neuromuscular disorder;
- A body mass index (BMI) > 30 kg/m^2^.

One of the aims of the used exclusion criteria was to create a homogenous test and control groups for decreasing the possible impact of patient individual variations on the results of the study. An additional reason for the BMI limit was the single-sized smart shirt prototype adopted in the study. Although the fitness shirt was quite elastic and could be used by people of different shapes, the textile stretch sensors are less sensitive when overstretched.

### 2.4. Intervention

#### 2.4.1. All Patients

All participants in the first visit were introduced to the study and were requested to fill the Disabilities of the Arm, Shoulder, and Hand (DASH) questionnaire [20]. Each patient was examined through a standard routine assessment and was informed about the condition of their shoulders, as well as received advice on correction of the posture in daily routine. An individual training plan for 8 weeks long rehabilitation session (2 visits per week) was created according to the latest scientific literature [8,9,10,11,26] and the experience of the physiotherapist. The training plan consisted of two parts, where the main focus of the first part (first 4 weeks of the training) was a gradual strengthening of the rotator cuff and scapula stabilizers [10,27], during which the patient had to regain sufficient control of scapular orientation during arm movement. In the second part of the training plan, muscle-specific strength exercises were introduced [9,26,27,28]. The exercises were performed under the supervision of the physiotherapist by using the visual feedback from the mirror, when necessary. During the movement control exercises, the scapular position was optimized to the thorax, initially by being altered manually by the therapist on a subject-specific basis. When required, the physiotherapist performed manual treatment by stretching the posterior glenohumeral capsule, as it has been demonstrated previously that posterior glenohumeral capsule stretch in addition to a conventional program is beneficial for patients with SAPS [29]. Training exercises were performed at a slow, controlled pace, with a strong accent on the control of the motion. Each visit, except for the first and last, lasted 30 min. In between these supervised sessions, participants were advised to follow the recommendations about posture corrections, but no guidelines for performing exercises at home were provided. During the last visit, the patient was requested to repeat the DASH questionnaire, as well as perform additional tests.

#### 2.4.2. DAid Smart Shirt Group

**Figure 4:**
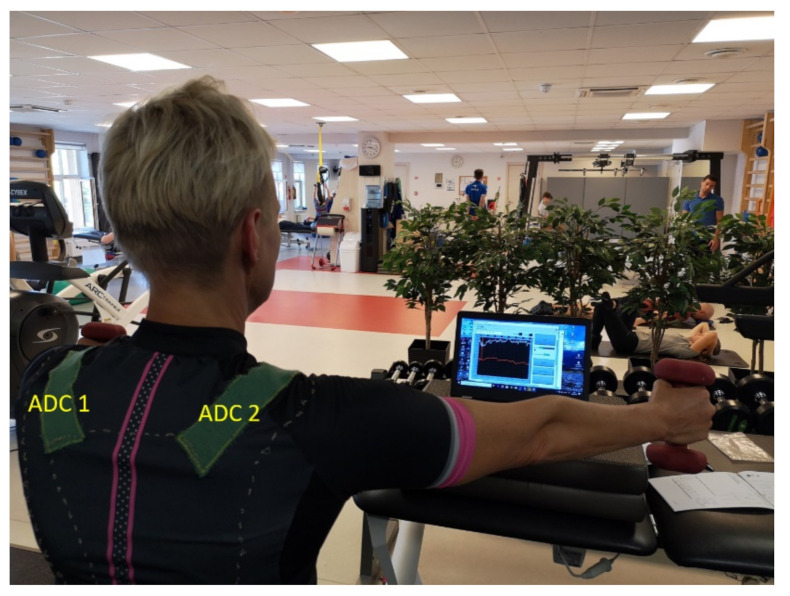
Application of the DAid system during a bilateral arm lift exercise.

**Figure 5:**
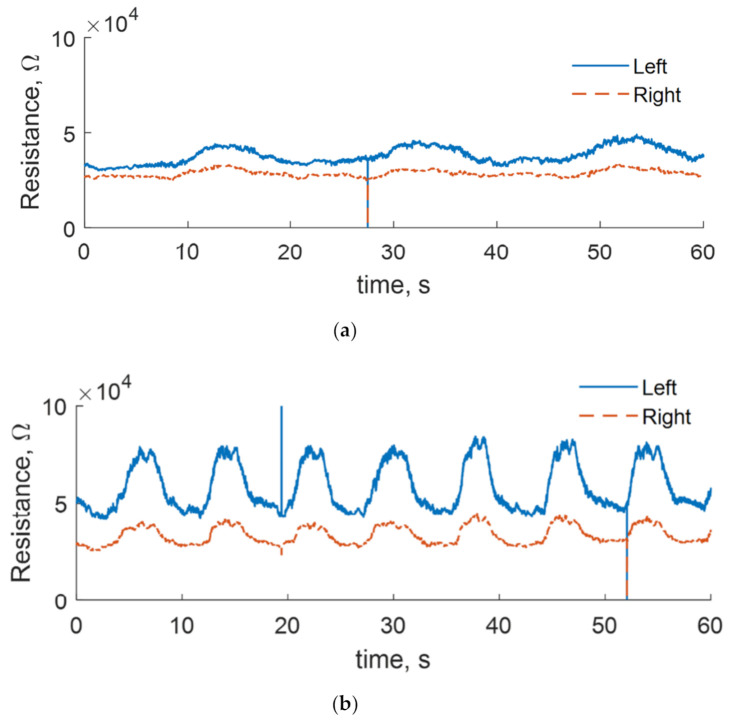
A raw measurement of the shoulder movement with the DAid shirt system for bilateral arm lift exercise. Comparison between the sensor reading for (**a**) a correct (slow, controlled) movement and (**b**) an incorrect, relatively fast movement with no control over the shoulder position. A considerably lower amplitude change during a controlled motion indicates less shoulder motion during the exercise.

During the first visit, the patients, who agreed to be included in the DAid smart shirt test group, were informed about the nature of the study and were instructed on the proper procedure of application of the smart garment during the training. The DAid system was put over the clothes of the patient, and a laptop was positioned in a comfortable height and position in front of the patient. The real-time reading from both sensors was provided to the patient in form of a graph on the computer screen (see Figure 4), and the participant was instructed to perform each exercise while actively trying to keep the sensor reading as even as possible. This could be achieved only through holding the shoulders immobile, as any shoulder motion would deform the shirt fabric and thus be visible on the screen as a change of sensor reading. In a perfect situation, performing an exercise with no motion of the shoulders would result in a close to a flat line on the sensor response, as the sensors would not be deformed. In most cases, patients are unable to perform the movement perfectly, resulting in a periodically changing value of the measured sensor resistance as the exercise is performed. Considering that the exercises in most cases were lifting and lowering one or both arms in a specific manner, the sensors were stretched and released periodically. Consequently, the relative change of the sensor value described the quality of the performed exercise (Figure 5). The patients were instructed to try to keep the change as low as possible. This forced the patients to pay more attention to the stability of the shoulder. The participants were under constant supervision of the physiotherapist.

### 2.5. Outcome Measures

Pre-treatment (baseline) and post-treatment assessments were performed for each participant. All visits were attended to by the same physiotherapist and standardized protocols were applied. Following shoulder function assessment tools were adopted post-treatment according to SAPS treatment guidelines [9,27]—the DASH score, CKCUES test, and external and internal rotation ratio in 90–0 and 90–90 positions. The DASH score is a measure of disability and symptoms for musculoskeletal disorders of upper limbs, in the form of a questionnaire. It requires the patient to provide a rating from 1 (no difficulty/symptoms/pain) to 5 (great difficulty/symptoms/pain) to a set of questions about performing various daily life activities. The CKCUES test is a physical test for evaluating the performance of the upper limbs in a closed kinetic chain by counting the number of times a participant is able to perform a specific exercise during a 15 s long period. The exercise in question is assuming a push-up position and touching the supporting hand with the swinging hand. This test is considered to be easy for physiotherapists to apply and for patients to understand due to no necessity for high technology. The external and internal rotation ratio is the ratio between the force exerted by external rotator (ER) and internal rotator (IR) muscles. The muscle strength for ER/IR ratio calculation was measured by a handheld dynamometer MicroFET^®^2 (Hoggan Health Industries Inc., Salt Lake City, UT, USA). The results of these tests were the main independent variables of the study.

Descriptive statistics were applied to present the baseline characteristics and functional test values, the mean, standard deviation (SD), and confidence interval (95% CI) were calculated across all subjects. The mean baseline characteristics and functional test values were compared between both groups using unpaired t-test and Mann–Whitney U test, as appropriate. All analyses were made within IBM SPSS Statistics V22.0 (IBM, New York, NY, USA).

## 3. Results

### 3.1. The Flow of Participants through the Study

**Figure 6:**
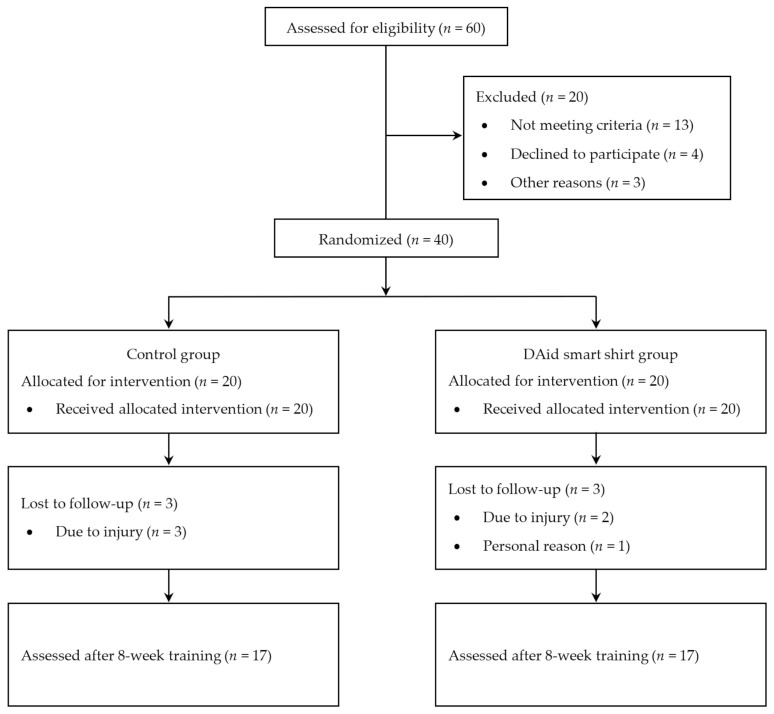
Flowchart of the study procedure.

Between January 2019 and January 2020, 60 patients with a diagnosis of primary subacromial pain syndrome (SAPS) were assessed for eligibility. The eligibility assessment was performed according to the Consolidated Standards of Reporting Trials (CONSORT) flow diagram, as presented in Figure 6. In total 40 participants met the inclusion criteria, of these 20 were assigned to the DAid smart shirt trial group and 20 to the control group. Six participants (three from each group) withdrew from the trial for various reasons, mostly (5/6) due to an injury, which prevented further participation in the training for a significant period. Complete test results for 34 participants were included in the statistical analysis.

The participant characteristics are provided in Table 1. Although the selection of participants for each group was randomized, quite a similar age and gender distribution were achieved in both groups. The mean age of participants in the test group and the control group was 38.6 (SD 12.6) and 40.8 (SD 10.1), respectively. The body mass index for the DAid smart shirt group and the control group was 22.0 (SD 1.5) and 23.1 (SD 1.0), respectively. For the same number (13/17) of participants in both groups, the dominant side was affected.

### 3.2. DASH Module Score Results

No statistically significant difference was detected between groups in the pre-intervention self-reported DASH score. The DASH general module scores were 54.4 (SD 2.5) for the test group and 52.0 (SD 4.3) for the control group. The DASH work module scores for the test group and the control group were 85.2 (SD 2.7) and 86.2 (SD 3.7), while the DASH sport/music module scores for the test group and the control group were 83.8 (SD 2.7) and 84.4 (SD 3.2), respectively. However, a significantly greater improvement (*p* ˂ 0.001) was observed for the DAid smart shirt group compared to the control group in the DASH score after 8 weeks of treatment. The mean DASH general module score for the test group was 14.6 (SD 3.1), while for the control group it was 21.5 (SD 3.0). For the DASH work module, the mean score for the test group was 17.1 (SD 3.3), while the control group score was 27.5 (SD 3.1). The mean DASH sport/music module scores were 18.1 (SD 3.3) and 25.7 (SD 2.9) for the test group and the control group respectively (see Table 2).

### 3.3. Shoulder Functional Performance Test Results

A statistically significant difference in closed kinetic upper extremity stability test results and isometric rotator cuff strength value ratio was detected between the test group and the control group after the intervention (see Table 3). The mean CKCUES test result at the end of the intervention for the test group was 22.2 (SD 3.9), while for the control group it was 18.1 (SD 3.0). The mean isometric strength ratio between the external and internal rotator muscles for the dominant (D) side in 90–0 position for the test group and the control group was 0.88 (SD 0.07) and 0.65 (SD 0.09), respectively, while for the non-dominant (ND) side, for the respective groups it was 0.89 (SD 0.06) and 0.62 (SD 0.10). In the 90–90 position, the mean ER/IR ratios for the test group were 0.74 (SD 0.14) and 0.77 (SD 0.11) for the dominant and non-dominant side, respectively, while for the control group the same results were 0.56 (SD 0.09) and 0.57 (SD 0.08).

## 4. Discussion

In this study, the DAid smart shirt system was applied in the rehabilitation of patients with SAPS for monitoring of scapular movements during motion control exercises and muscle-specific exercises. It was confirmed that patients, who performed the exercises with the feedback system, experienced significantly greater improvements in shoulder function after 8 weeks of rehabilitation compared to the control group. The decrease of pain and physical impairment during daily and work activities post-intervention was evaluated through the DASH questionnaire. The obtained post-treatment DASH scores for both study groups were significantly lower than the pre-treatment scores and comparable to those reported in the literature for similar studies about SAPS (9.64 ± 8.38, 95% CI 0.83–20.83) [20]. On top of that, the scores for the DAid smart shirt group were considerably lower than the scores of the control group, thus demonstrating the significance of visual feedback for rehabilitation training. The DASH scores for both post-treatment groups were close to the typical values for healthy individuals in the general population (10.10 ± 14.68 for DASH general) [30].

The achieved results for the CKCUES test for the test group, which used the DAid smart shirt system, were comparable to those of a sedentary and active healthy population (reference values 18.5 and 20.5 for males and females, respectively) [21]. The results for the control group were noticeably lower than those for the test group; however, the practical significance of the observed statistical difference is a matter of debate. A similar result was provided by the ER/IR ratio test, where the results from the test group were closer to those from a healthy population given in the literature [22], while the control group results were notably lower.

To fully benefit from the application of the feedback from the DAid system, a clear understanding of the reasons for the positive result is desirable. When compared to the conventional training method with a mirror as the source of the feedback, two main differences can be recognized, (1) the quality of the feedback and (2) the patient involvement. First, the textile sensors due to the position and sensitivity can detect minor movements that might be hard to distinguish in the mirror visually. Additionally, the simple line graph feedback gives the user an easy to comprehend and focus on the information about the movement quality, when, on the other hand, the increased amount of visual information in the case of the feedback from a mirror could negatively impact the quality of the training due to the limited visual attention [31]. Secondly, the interactive nature of digital feedback could play a role through the increased involvement of the patient during the training [14]. The result of a correctly performed movement, or rather the absence of a mistake, is immediately notable with the digital feedback from the sensors, and, thus, could keep the patient more focused during the whole training. These two aspects deserve a closer investigation in any subsequent study.

Besides the obvious health benefits, a physiotherapy training assistance system such as the DAid smart shirt has other possible advantages that should be mentioned. This research indicated that patients were capable of training with limited supervision by using the feedback from the system, thus removing the necessity for constant attention from a physiotherapist. This in turns could create an opportunity for a physiotherapist to work with several patients at once. Additionally, after the first few sessions, some patients should be able to perform the prescribed exercises with the feedback system under home conditions, thus greatly increasing the number of possible training sessions and, consequently, improving the recovery time. Application of such systems has a great potential for telerehabilitation and can be crucial for cases when patients due to various reasons are unable to visit the doctor routinely.

### Limitations

Although an overall positive result was achieved in this study, the likelihood of a certain bias has to be discussed. Considering the nature of the study, it could be argued that participation in the study itself might have an impact on the motivation of the participants, thus creating a positive effect on the result, which is unrelated to the application of the system. Unfortunately, for the present study, it was practically impossible to design a blinded trial, where the patients would use the system without knowing it. A blinded study where the control group would be provided with simulated sensor feedback could be potentially harmful because a patient with an incorrect movement pattern would receive feedback indicating a correct movement. The true benefit of the system might be clear only after the application of such systems has become more widespread, thus the patients will be more accustomed to the system, removing the effect of ‘novelty’.

A single size prototype was used during the study for all patients. Although the participants formed a rather uniform group, the same shirt could not have the same fit for all patients. Depending on the fit, the pre-strain of sensors could vary, thus leading to interpatient variation or even a lack of sensors’ sensitivity. Ideally, each patient should have an individual, size-fitted garment for maximal performance.

The system has several issues that affect its robustness and require attention during application. First, the shirt has to be readjusted before each exercise. Although readjusting involves simply pulling the shirt down in case the upward lift of arms during an exercise has lifted the shirt, failing to do so could decrease the sensitivity of the sensors. The second issue is the influence of sweating on the performance of the textile stretch sensors. When the garment reaches certain wetness, the skin under the sensors starts to act as a conductor instead of the sensors, thus considerably lowering the sensitivity. In the present study, this issue was not observed as the participants were wearing the smart shirt on top of their clothes to avoid soaking it with sweat during the session. Moreover, rehabilitation training, in general, is a lower intensity training and thus does not induce as much sweating compared to a normal strength training.

## 5. Conclusions

The impact of the application of a smart garment system on the outcome of rehabilitation training for SAPS patients was evaluated. A clinical study was performed where patients with subacromial pain syndrome were training with the assistance of the feedback from the DAid smart shirt. The smart garment system detects undesirable movements of the shoulder, which could be difficult to visually detect in the mirror; thus, allowing the user to adjust movements during the training. The clinical study results demonstrated that the application of such a system significantly improved the recovery of patients with SAPS. To our knowledge, this is the most extensive study of a purely textile-based smart garment application for physiotherapy training assistance for SAPS patients under the clinical environment. The results of this study should encourage the application of similar systems in physiotherapy and rehabilitation, especially in the context of telemedicine.

## Figures and Tables

**Table 1:** Characteristics of participants.

Characteristics	DAid Smart Shirt Group (*n* = 17)	Control Group (*n* = 17)
Age (years)	38.6 ± 12.6	40.8 ± 10.1
Sex (Male/Female)	7/10	8/9
Body mass index (kg/m^2^)	22.0 ± 1.5	23.1 ± 1.0
Dominant side affected	13 (76.5%)	13 (76.5%)

**Table 2:** The Disabilities of the Arm, Shoulder, and Hand (DASH) pre- and post-treatment score, mean, SD, and CI.

	DAid Smart Shirt Group	Control Group
DASH General		
Baseline	54.4 ± 2.5 (95% CI 53.2 to 55.7)	52.0 ± 4.3 (95% CI 49.8 to 54.2)
8th week	14.6 ± 3.1 (95% CI 13.1 to 16.2)	21.5 ± 3.0 (95% CI 20.0 to 23.1)
DASH Work		
Baseline	85.2 ± 2.7 (95% CI 83.8 to 86.6)	86.2 ± 3.7 (95% CI 84.3 to 88.1)
8th week	17.1 ± 3.3 (95% CI 15.3 to 18.8)	27.5 ± 3.1 (95% CI 25.9 – 29.1)
DASH Sport/music		
Baseline	83.8 ± 2.7 (95% CI 82.4 to 85.2)	84.4 ± 3.2 (95% CI 82.7 to 86.0)
8th week	18.1 ± 3.3 (95% CI 16.4 to 19.8)	25.7 ± 2.9 (95% CI 24.2 to 27.2)

**Table 3:** Shoulder functional test post-treatment results (mean SD, and CI) for Closed Kinetic Chain Upper Extremity Stability (CKCUES) test and internal/external (ER/IR) rotation ratio of the dominant (D) and non-dominant (ND) side.

	DAid Smart Shirt Group	Control Group
CKCUES test	22.6 ± 3.9 (95% CI 20.2 to 24.2)	18.1 ± 3.0 (95% CI 16.6 to 19.7)
ER/IR ratio (90–0) D	0.88 ± 0.07 (95% CI 0.85 to 0.92)	0.65 ± 0.09 (95% CI 0.60 to 0.70)
ER/IR ratio (90–0) ND	0.89 ± 0.06 (95% CI 0.86 to 0.93)	0.62 ± 0.1 (95% CI 0.57 to 0.67)
ER/IR ratio (90–90) D	0.74 ± 0.1 (95% CI 0.67 to 0.81)	0.56 ± 0.09 (95% CI 0.51 to 0.61)
ER/IR ratio (90–90) ND	0.77 ± 0.1 (95% CI 0.72 to 0.83)	0.57 ± 0.08 (95% CI 0.53 to 0.61)
